# Switching Roles: Beneficial Effects of Adipose Tissue-Derived Mesenchymal Stem Cells on Microglia and Their Implication in Neurodegenerative Diseases

**DOI:** 10.3390/biom12020219

**Published:** 2022-01-27

**Authors:** Ana Isabel Sánchez-Castillo, M. Rosario Sepúlveda, José Luis Marín-Teva, Miguel A. Cuadros, David Martín-Oliva, Elena González-Rey, Mario Delgado, Veronika E. Neubrand

**Affiliations:** 1 Department of Cell Biology, Faculty of Sciences, University of Granada, Av. Fuentenueva, 18071 Granada, Spain; anabelsc02@correo.ugr.es (A.I.S.-C.); mrsepulveda@ugr.es (M.R.S.); jlmarin@ugr.es (J.L.M.-T.); macuadro@ugr.es (M.A.C.); dmoliva@ugr.es (D.M.-O.); 2 Department of Cell Biology and Immunology, Institute of Parasitology and Biomedicine López-Neyra, IPBLN-CSIC, Av. Conocimiento 17, Parque Tecnológico de la Salud, 18016 Granada, Spain; elenag@ipb.csic.es (E.G.-R.); mdelgado@ipb.csic.es (M.D.)

**Keywords:** adipose tissue-derived mesenchymal stem cells, extracellular vesicles, microglia, neurodegenerative diseases, neuroinflammation, neuroprotection

## Abstract

Neurological disorders, including neurodegenerative diseases, are often characterized by neuroinflammation, which is largely driven by microglia, the resident immune cells of the central nervous system (CNS). Under these conditions, microglia are able to secrete neurotoxic substances, provoking neuronal cell death. However, microglia in the healthy brain carry out CNS-supporting functions. This is due to the ability of microglia to acquire different phenotypes that can play a neuroprotective role under physiological conditions or a pro-inflammatory, damaging one during disease. Therefore, therapeutic strategies focus on the downregulation of these neuroinflammatory processes and try to re-activate the neuroprotective features of microglia. Mesenchymal stem cells (MSC) of different origins have been shown to exert such effects, due to their immunomodulatory properties. In recent years, MSC derived from adipose tissue have been made the center of attention because of their easy availability and extraction methods. These cells induce a neuroprotective phenotype in microglia and downregulate neuroinflammation, resulting in an improvement of clinical symptoms in a variety of animal models for neurological pathologies, e.g., Alzheimer’s disease, traumatic brain injury and ischemic stroke. In this review, we will discuss the application of adipose tissue-derived MSC and their conditioned medium, including extracellular vesicles, in neurological disorders, their beneficial effect on microglia and the signaling pathways involved.

## 1. Introduction

Many neuropathologies, including neurodegenerative diseases such as Alzheimer´s disease (AD) and Parkinson´s disease (PD), traumatic brain injury (TBI), spinal cord injury (SCI) and ischemic stroke, among others, are characterized by neuroinflammation. All these pathologies have in common a disturbance in brain homeostasis that frequently results in neuronal cell death, although the initiator of this imbalance is different in each case. They range from the formation of extra- or intracellular protein aggregates, in the case of AD and PD, to lesions in TBI and SCI, and to alteration of the blood supply in ischemic stroke. Moreover, the various brain areas can be differentially affected. Thus, while AD patients exhibit β-amyloid (Aβ) senile plaques and intraneuronal neurofibrillary tangles of hyper-phosphorylated tau in the entorhinal cortex and hippocampus [1], PD is instead characterized by α-synuclein aggregations and the cell death of dopaminergic neurons in the substantia nigra (SN) [2]. In contrast, lesions provoked by ischemic stroke can affect any brain region. In spite of different locations and lesion types, all these neuropathological insults share a similar outcome, namely the pro-inflammatory activation of microglia, which represent the immune cells of the central nervous system (CNS) and are its first line of defense [3]. This activation leads to the microglial secretion of a variety of neurotoxic factors, such as pro-inflammatory cytokines and chemokines, as well as reactive oxygen species (ROS) or nitric oxide (NO) [4]. In addition, the blood-brain barrier becomes leaky and immune cells from the periphery can infiltrate the CNS, enhancing the inflammatory environment [5].

From a therapeutic point of view, this neuroinflammation has to be mitigated to prevent further neuronal loss and cognitive disorders. To this end, mesenchymal stem cells (MSC) as anti-inflammatory agents have been applied in a variety of pre-clinical and clinical studies with promising results [6,7,8,9,10,11]. MSC are adult stem cells with the potential to differentiate into cells of the mesenchymal lineage [12]. However, their use in inflammatory diseases is rather based on their immunomodulating properties.

Since MSC were first isolated from bone marrow [13], many studies so far rely on bone marrow MSC (BM-MSC). However, MSC can also be obtained from different adult tissues, such as adipose tissue, liver, dental pulp, endometrium, muscle, amniotic fluid, placenta and umbilical cord blood [12]. In particular, MSC from adipose tissue (ASC) have become a valuable therapeutic tool, due to their availability and easy extraction methods. Recently, their use in neurological disorders with underlying neuroinflammation by chronic microglial activation has emerged as a powerful treatment in animal models. Therefore, in this timely review, we would like to provide an overview of the positive effect of ASC on microglia functions, among others on the activation of the neuroprotective PI3K/Akt/RhoGTPase signaling pathway. Consequently, microglia can play a beneficial role in a wide spectrum of neuropathologies.

## 2. Role of Microglia in Health and Disease

In the healthy brain, microglia are continuously surveilling their environment [14], maintaining CNS homeostasis. They can secrete neurotrophic factors and clear up cellular debris in response to brain alterations, thus playing an active CNS-supporting role [15,16]. However, upon injury, stroke or certain chronic stimuli in the brain, they can become excessively reactive and change toward a detrimental inflammatory phenotype. This plastic nature of microglia has led to the identification of many different activity states of these cells [15,17,18], supported later by gene expression data [19]. As this review will not focus on the different phenotypes of microglia, for simplification we will use the terms “pro-inflammatory” and “neuroprotective” herein in order to distinguish between the two main activity states, which is essential to understand the effects of ASC on microglia.

**Figure 1:**
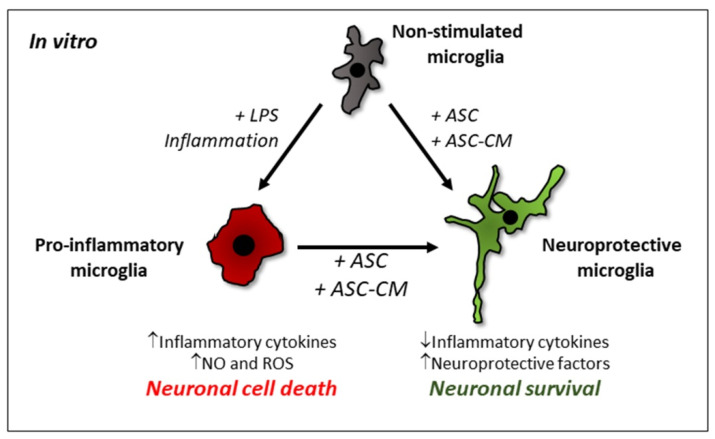
Simplified view of microglia phenotypes modulated by adipose tissue-derived mesenchymal stem cells (ASC) or their conditioned medium (ASC-CM) in vitro. Non-stimulated microglia cultured in growth medium under standard conditions (grey) can switch to a pro-inflammatory (red) or neuroprotective (green) phenotype, depending on their stimulatory triggers. In general, the pro-inflammatory phenotype is characterized by a more amoeboid cell shape and the secretion of neurotoxic substances, such as inflammatory cytokines, nitric oxide (NO) and reactive oxygen species (ROS). ASC or ASC-CM can also revert a pro-inflammatory microglial phenotype into a neuroprotective one, acquiring a ramified morphology, decreasing the expression of inflammatory cytokines and secreting neuroprotective factors.

Figure 1 schematically depicts the induction of these phenotypes under different stimuli in vitro. The possibility of switching from pro-inflammatory to neuroprotective microglia actually makes these cells an attractive therapeutic target in neuroinflammatory diseases, as it is a way to influence their inflammatory state, trying to keep them in a neuroprotective, CNS-supporting role. Very interestingly, this change can be followed morphologically in vitro (Figure 1), as the neuroprotective phenotype has a predominant elongated cell shape with some ramifications, while the pro-inflammatory phenotype is mostly associated with a rounded morphology [16]. The pro-inflammatory state can be provoked by different triggers, e.g., by lipopolysaccharide (LPS), but also by Aβ (a hallmark of AD) or α-synuclein (a hallmark of PD) [1,4]. This phenotype is associated with the secretion of pro-inflammatory cytokines, such as tumor necrosis factor-α (TNF-α), interleukin (IL)-1β, IL-6, and ROS, as well as NO, leading ultimately to neuronal cell death [4]. The switch toward the neuroprotective phenotype has been less extensively studied and can be induced, for example, by IL-4 [20] and MSC [10,21,22]. The upregulation of neuroprotective molecules, such as brain-derived neurotrophic factor (BDNF), activity-dependent neurotrophic protein (ADNP) and the fractalkine receptor CX3CR1, characterizes this phenotype [21,22], as well as the expression of arginase-1 [10,22], a marker for alternatively activated macrophages [23] and for anti-inflammatory microglia [24].

Nowadays, the aim of treatment in many neuropathological events with microglial involvement is to reduce the pro-inflammatory phenotype, shifting the balance toward a neuroprotective one in order to reduce neuronal cell death. Indeed, in the last decade, ASC administration both in vitro and in vivo has been shown to reach this goal, as will be summarized below.

## 3. Origin and Characteristics of Adipose Tissue-Derived Mesenchymal Stem Cells (ASC)

ASC are considered a heterogeneous cell population from the stromal fraction of adipose tissue (such as abdominal fat, infrapatellar fat pad, and buccal fat pad) that can be easily obtained by collagenase digestion of the isolated tissue and shows strong adherence to plastic under culture conditions. They express specific cell surface markers, including CD29, CD44, CD73, CD90, CD105, CD146 and CD166, while lacking the expression of CD11b, CD14, CD19, CD31, CD34, CD45 and CD79a [25]. Moreover, the fact that ASC express low levels of human leukocyte antigen (HLA) class I molecules, and do not express HLA class II molecules or co-stimulatory molecules, such as CD40, CD40L, CD80 and CD86, confers them an “immune-privileged” state that allows them to escape from the cytotoxic effects of lymphocytic T cells, B cells and natural killer (NK) cells. As other MSC, ASC show self-renewal and multi-differentiation potential, with the capacity to generate chondrocytes, osteoblasts and adipocytes [25]. Besides their regenerative capacity, ASC possess immunomodulatory and anti-inflammatory effects, making them attractive candidates for therapeutic use in various diseases involving immune dysregulation and extensive tissue damage, including graft-versus-host disease, rheumatoid arthritis, Crohn’s disease, sepsis and COVID-19, among others [26,27]. Preclinical experimental models indicate that ASC exert their immunoregulatory activity by various non-exclusive and redundant mechanisms that involve the secretion of a plethora of anti-inflammatory mediators, the suppression of T cell-mediated self-reactive responses and the induction of immune tolerance by re-educating immune cells, i.e., regulatory T cells, tolerogenic dendritic cells, alternatively activated macrophages and microglia [6,28,29,30,31]. Some of these effects require cell-to-cell contact, while others are mediated in a paracrine manner through soluble mediators. These findings have opened up the discussion about the convenience of therapies based on ASC or their cell products, such as their conditioned medium (CM) or extracellular vesicles (EV). At this moment, therapies that use ASC have adequately progressed to clinical practice; however, the appropriateness of treatments that are based on their products are still under debate until the nature of their components are fully defined and the methods for their isolation are standardized [32,33,34]. In this sense, it exists a wide variety of mediators that have been identified by different research groups in ASC-derived CM, with critical regulatory factors that are present in some studies but absent in others; undoubtedly, this is a research area of high interest.

## 4. Effect of ASC on Microglia Phenotype In Vitro

MSC in general exert immune-modulatory and anti-inflammatory effects on a variety of cell types [12,35,36]. This was also demonstrated for microglia, using MSC of different origins, including ASC [7,8,9,10,21,22,37,38,39].

**Figure 2:**
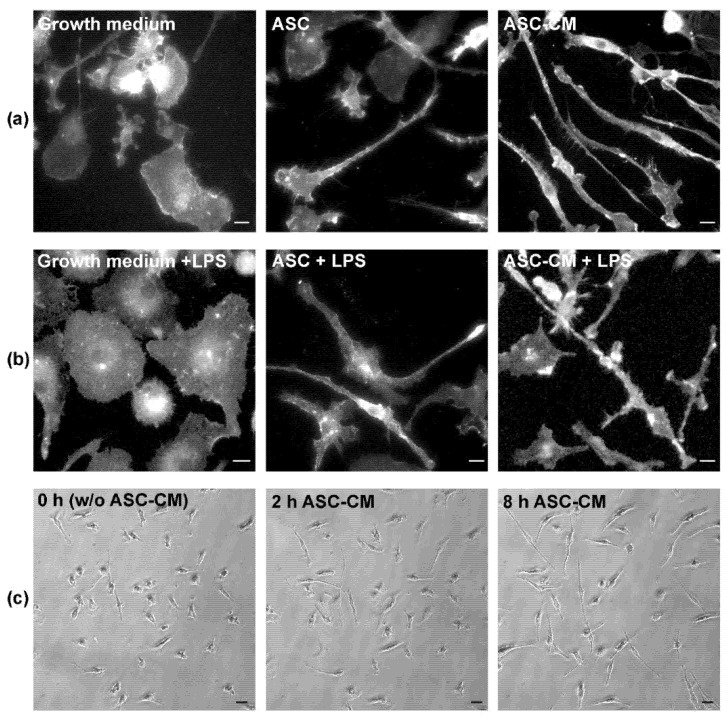
Microglia ramification upon incubation with adipose tissue-derived mesenchymal stem cells (ASC) or their conditioned medium (ASC-CM). (**a**) Mouse ASC were seeded in transwell plates and subsequently transferred to wells containing murine primary microglia. After 48 h, the microglia were fixed, immunostained for CD11b and pictures were taken on a Zeiss Axiophot fluorescence microscope with a 40× objective. Alternatively, microglia were incubated for 48 h with ASC-CM and then treated as described before. In both conditions, they acquired an elongated morphology, compared with microglia incubated only with growth medium. (**b**) Even in the presence of lipopolysaccharide (LPS), which provoked an amoeboid cell shape, ASC and ASC-CM induced microglial ramification. A detailed description of all materials and methods used for these experiments can be found in [22]. White scale bars = 10 μm. (**c**) Primary murine microglia were plated in a 12-well plate in microglia growth medium, as described in [22]. After three days, they were subjected to live-cell imaging on a Leica DM IRB HC FLUO widefield microscope equipped with a digital camera (DFC 300 FX) and a plate incubation system with a temperature (37 °C) and CO_2_ control (5% CO_2_), using a 20× objective suitable for contrast-phase microscopy. Immediately after the 0 h time point, ASC-CM was added to the cells and pictures were taken every 10 min from the same field of view. After 2 h, the microglia started to elongate and were ramified by the 8 h time point. The time-lapse video is available in the supplementary material as Supplementary Video S1. Black scale bars = 100 μm. Images from data not shown and replicates from [22].

One of the first studies incubating ASC with primary mouse microglia in transwell inserts in vitro has shown that primary mouse microglia drastically change their morphology into an elongated cell shape ([22]; Figure 2A, middle panel). As these inserts preclude direct cell-to-cell contact, soluble factors must be responsible for this morphology change. Indeed, the conditioned medium from ASC (ASC-CM) also induces this cell shape (Figure 2A right panel and C and Supplementary Video S1), indicating that the soluble factors of ASC induce microglia ramification. In fact, after 2 h of incubation with ASC-CM, the microglia started to elongate, reaching a high degree of elongation after 8 h (Figure 2C and Supplementary Video S1). As the ASC-CM is routinely filtered with a pore size of 0.2 μm prior to its incubation with microglia, the involvement of exosomes, which are about 30–120 nm in size [35], or other small-sized EV cannot be excluded in the induction of this phenotype (see Section 6.2. “Application of ASC-Generated Extracellular Vesicles (EV) In Vivo”).

As mentioned above, primary microglia in vitro acquire a rather round cell shape under inflammatory conditions, e.g., when stimulated with LPS (Figure 2B left panel), while in vivo, in pathological events of the CNS, an amoeboid microglial morphology and sometimes a large soma with several processes are observed [16,40]. The anti-inflammatory effect of ASC in transwell inserts or of ASC-CM is easily detectable by microscopy, as rounded pro-inflammatory cells acquire an elongated shape in the presence of ASC in transwell inserts or ASC-CM (Figure 2B, middle and right panel, respectively). More strikingly, ASC-CM also reverts an inflammatory phenotype, induced by 24 h pre-incubation with LPS prior to ASC-CM addition, to a neuroprotective phenotype [22]. The latter observation is of special importance, as this might be extrapolated to the in vivo studies using ASC, when mice already suffering a neurological disease are injected with ASC and recover at least partially from their symptoms (see Section 6. “Application of ASC In Vivo”).

This morphological change of microglia in vitro goes along with changes in the expression levels of pro-inflammatory cytokines and neuroprotective factors. The expression of the LPS-induced pro-inflammatory cytokines TNF-α and IL-6 are reduced by ASC-CM, while neuroprotective factors, such as BDNF, ADNP and fibroblast-growth factor (FGF)-2, are increased [22]. In addition, arginase-1, a marker for alternatively activated macrophages [23], and phagocytosis, which is another feature of neuroprotective microglia [24], are increased by ASC or their ASC-CM [22]. Remarkably, a phagocytic event executed by the upper-left cell was imaged in Supplementary Video S2. The phagosome, identified as a white spot inside the cell, remains visible until nearly the end of the video. The increase in the phagocytic rate is of special therapeutic interest since a decrease in phagocytosis was associated with increasing Aβ plaques in a mouse model for AD [41].

Later on, these neuroprotective effects of ASC were also observed in the microglial murine cell line BV-2 [7,8,42]. However, the authors of these studies used slightly modified ASC or ASC-CM: BV-2 cells were incubated with an ASC-concentrated conditioned medium (ASC-CCM) from cells pre-stimulated with inflammatory cytokines [8]. This pre-stimulation with TNFα and IL-1β led to the expression and secretion of the anti-inflammatory TNF-stimulated gene 6 protein (TSG-6), as shown previously with BM-MSC [37]. Subsequently, the anti-inflammatory properties of non-pre-stimulated ASC-CCM were compared to those of pre-stimulated ASC-CCM by measuring the nitrite concentration in the microglia supernatant. While both media, ASC-CM and ASC-CCM, decreased the LPS-induced NO production, the pre-stimulated ASC-CCM was more effective. Similarly, LPS and the interferon-γ (IFN-γ)-induced expression of CD86 and IL-1β, both pro-inflammatory markers of microglia, were reduced when treated with pre-stimulated ASC-CCM, while arginase-1 expression was increased. At the same time, Iba-1 staining, which is increased upon LPS stimulation [43], was reduced by ASC-CCM. In addition, BV-2 cells changed from a rounded morphology when treated with LPS and IFN-γ, to an elongated morphology upon ASC-CCM addition [8], although this conversion is not as prominent as that observed with primary microglia (Figure 2). All these anti-inflammatory effects were reverted, when TSG-6 in ASC and, subsequently, in their CCM, was efficiently downregulated by siRNA, indicating a key role for this protein in the ASC secretome and its anti-inflammatory features [42].

Similarly, Huang and colleagues downregulated the transcription factor Nrf2 by siRNA in ASC, leading to a decrease of heme oxygenase-1 (HO-1) expression levels, a protein involved in redox homeostasis in cells that is known for its important antioxidant activity [44]. Subsequently, the authors co-cultured these modified cells with BV2-cells and showed that the ASC-induced anti-inflammatory effect on BV-2 cells was diminished by this modification, pointing towards a crucial role of Nrf2/HO-1 signaling in the anti-inflammatory properties of ASC [7].

In summary, ASC and their CM cause a switch in microglia toward a neuroprotective phenotype, identified by cell elongation in vitro, a decrease in the release of pro-inflammatory cytokines, and the induction of neuroprotective and anti-inflammatory factors. Nrf2/HO-1 and TSG-6 expression in ASC seem to play a key role in acquiring this microglial phenotype.

## 5. ASC-Induced Activation of the PI3K/Akt/RhoGTPase Signaling Pathway in Microglia

As we have seen in the previous section, there is no doubt that ASC are capable of inducing a neuroprotective phenotype in microglia. This conclusion, however, opens up the question of which signaling pathways in microglia are involved in acquiring this phenotype. Proteins taking part in these pathways might represent novel drug targets, as by activating or inhibiting them they could shift microglia from a pro-inflammatory to an anti-inflammatory neuroprotective state.

Interestingly, CSF-1 has been detected in the ASC-CM (unpublished data from M.D.) and the loss of its receptor (CSF-1R) in mice results in the ablation of nearly all microglia [45], representing an important initiator of microglia survival pathways. In line with these observations, ASC-CM containing anti-CSF-1 antibodies inhibited microglia ramification in vitro [22]. This result excludes the possibility of IL-34 as a CSF-1R ligand in the CM in these experiments, although IL-34, by binding to CSF-1R, plays a similar role as CSF-1R in microglial survival in vivo. [46].

Downstream targets of the CSF1-R in macrophages are, for example, phosphoinositol-3-kinase (PI3K) and protein kinase B (PKB)/Akt and have been reviewed on many occasions [47,48]. In primary microglia, this survival pathway is very likely to be implicated in microglia ramification induced by ASC-CM, as (i) Akt is strongly phosphorylated by ASC-CM, and (ii) the PI3K inhibitor LY 294002 reverses the ASC-CM-induced ramification [22]. On the contrary, although the extracellular-signal-regulated kinase 1/2 (Erk1/2), as CSF1-R downstream target, is strongly phosphorylated in microglia stimulated by ASC-CM, its inhibition by the MEK inhibitor PD 98059 does not counteract ASC-CM-induced microglial ramification [22].

**Figure 3:**
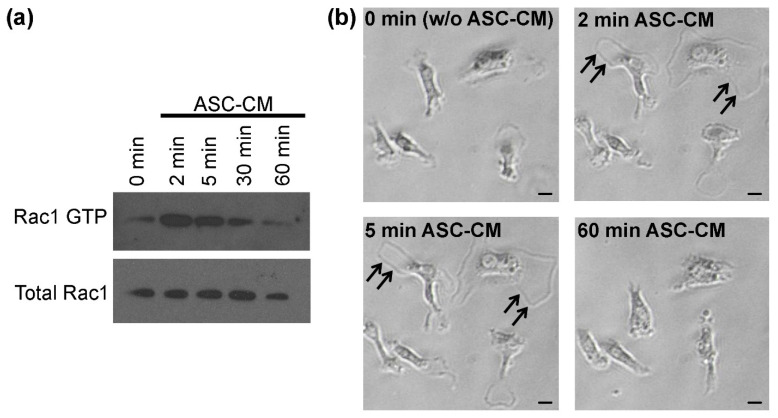
Rac1 activation and lamellipodia formation upon the administration of conditioned medium from adipose tissue-derived mesenchymal stem cells (ASC-CM). (**a**) Primary murine microglia were incubated with ASC-CM until the indicated time points, then they were harvested and subjected to a Rac1 activation assay (see [22] for a detailed description of the materials and methods). At the 2 min and 5 min time points, Rac1 was detected in its GTP-bound activated form. Western blot republished with permission of JOHN/WILEY & SONS, INC., from [22]; permission conveyed through Copyright Clearance Center, Inc. (**b**) Primary murine microglia were plated in a 12-well plate in microglia growth medium and subjected to live-cell imaging as described in Figure 2C. Immediately after the 0 min time point, ASC-CM was added to the cells and pictures were taken every min from the same field of view. At the 2 min and 5 min time points, lamellipodia formation was clearly visible at the edges of the cells (arrows). After 60 min, Rac1 activation (**a**) and lamellipodia formation (**b**) had stopped. Scale bars = 20 μm. Images from data not shown and replicates from [22].

Once PI3K is activated, it generates phosphoinositol-3-phosphate (PIP3), to which pleckstrin-homology (PH) domains possess a strong affinity [49]. In the Dbl-family of Rho guanine nucleotide exchange factors (RhoGEFs), Dbl homology (DH) domains are associated with PH domains, which assist in RhoGTPase activation [50]. Among the latter ones are the small RhoGTPases, RhoA, Rac1 and Cdc42, the main regulators of the actin cytoskeleton, which is ultimately responsible for morphological changes in any cell type. Similar to macrophages, where CSF-1R signaling has previously been linked to Rac1 activation [51], the transfection of dominant-negative mutants of Rac1 and Cdc42 in primary microglia efficiently inhibits ASC-CM-induced ramification. Furthermore, ASC-CM strongly activates Rac1 and Cdc42 in microglia [22] (Figure 3A). As described on many occasions, Rac1 induces the formation of lamellipodia [52,53], the mesh-like polymerization of F-actin. These structures are easily detectable by contrast-phase microscopy as a rim along the cell edges. As observed by live-cell imaging, lamellipodia are formed in primary microglia (arrows in Figure 3B and Supplementary Video S2) at the same time points of major Rac1 activation, namely, at 2 min and 5 min, as confirmed by biochemistry (Figure 3A). After 60 min, Rac1 activation and lamellipodia formation completely diminish (Figure 3). In line with these results, Rac1 downregulation by siRNA reduces ASC-CM-induced microglial ramification, indicating an essential role of Rac1 in this process [54].

But not only ASC use the PI3K/Akt/RhoGTPase pathway in order to induce a neuroprotective phenotype, also human umbilical MSC exert anti-inflammatory effects on microglia, such as the reduction of inflammatory markers and microglia ramification, and activate Akt, Rac1 and Cdc42. In fact, the MSC-induced increase in phagocytosis is inhibited by the PI3K inhibitor, LY 294002 [55].

Because ASC-CM contains a mixture of many different components (unpublished data from M.D.) [56,57], there might also be other signaling molecules and pathways involved in the switch to a neuroprotective microglial phenotype, leading to synergistic effects with the pathway described above. In order to shed light on genes implicated in these pathways, Neubrand and colleagues performed an siRNA screen of primary microglia stimulated with ASC-CM to induce microglial ramification [54]. The siRNA-downregulated expression of certain genes significantly inhibits microglial ramification. Consequently, these genes might play a relevant role in conversion to a neuroprotective phenotype. Out of these 45 hits, seven genes were validated (RhoE/Rnd3, Tiam1, p38β/Mapk11, GM-CSFR/Csf2ra, Map3k2, Creb1 and IκBα/Nfkbia) that are all potentially involved in ASC-CM-induced neuroprotective signaling. For example, GM-CSFR, the receptor for GM-CSF, might initiate the signaling cascade. In line with this, GM-CSF, when administrated to a PD mouse model, decreases microglial activation and increases dopaminergic neuron survival [58], indicating a neuroprotective role for its receptor. Further downstream might act the MAP kinase p38β and the transcription factor Creb1; interestingly, the downregulation of either one also inhibits ASC-CM-induced BDNF production [54]. As changes in microglial morphology are related to rearrangements of the cytoskeleton, the RhoGEF Tiam1 and the small RhoGTPase RhoE might be implicated in the cell shape change. Indeed, the downregulation of both of them enhances microglial cell migration [54], a process strongly dependent on the actin cytoskeleton. Interestingly, Tiam1 possesses two PH domains [59] that can bind to PIP3 generated by PI3K and might take part in the PI3K/Akt pathway described above.

While the latter findings are rather speculative, the PI3K/Akt/RhoGTPase pathway in microglia upon MSC stimulation is more established [22,55] but is likely not to be the only one.

## 6. Application of ASC In Vivo

Because of the immunomodulatory properties of MSC, their easy generation and availability, the lack of ethical concerns and of immune rejections, they are the ideal tools to downregulate neuroinflammation in a variety of neurodegenerative diseases. Therefore, their application has been tested in diverse pre-clinical animal models with encouraging results. In this section, we will concentrate on the findings of the beneficial actions of ASC, while the effect of MSC from other sources, e.g., of bone marrow or umbilical origin, are described elsewhere [11,35,60]. There are three ways to administrate ASC in vivo: (i) transplantation of cells, (ii) injection of ASC-CM, or (iii) injection of ASC-EV.

When initial animal studies with ASC were conducted, it was not entirely clear whether the neuroprotective effects were derived from the engrafted ASC themselves, their differentiation into neuronal cells, or a paracrine effect by secreting anti-inflammatory and neuroprotective factors. From experiments comparing in vitro expanded naïve and differentiated ASC, which were transplanted into the 6-hydroxydopamine mouse model for PD, it was concluded that the differentiation of ASC into neuronal cells was not necessary to exert neuroprotective effects, which were instead achieved by the secretion of trophic factors [61]; this was in agreement with the in vitro data using ASC-CM, as described in above. Thus, we decided to summarize the data from (i) cell transplantation and (ii) the injection of ASC-CM in one section, although in both administration methods, we cannot exclude the possibility that the therapeutic effect might be achieved by secreted biologically active EVs. As the administration of ASC-EV (iii) is an upcoming therapeutic strategy and requires detailed explanations, we dedicated an entire section to this topic.

### 6.1. Application of ASC In Vivo by Cell Transplantation and ASC-CM Injection

In general, microglia in vivo acquire a neuroprotective phenotype upon ASC or ASC-CM administration, with the result of lowering damage-inducing neuroinflammation in a variety of animal models and ameliorating disease-associated symptoms [7,8,42,57,62]. For example, Ma and co-workers transplanted ASC into the hippocampus of the Alzheimer Aβ precursor protein (APP)/Presilin1 (PS1) mice [62], a double transgenic model for AD with mutations in APP and PS1. These mice present cerebral Aβ plaques, which increase with age and are surrounded by glial inflammation. Interestingly, the Aβ burden in ASC-transplanted mice is significantly reduced, associated with the fact that microglia found in the vicinity of Aβ plaques in ASC-transplanted animals are labeled with IL-4, which induces alternatively activated macrophages [63] and a neuroprotective phenotype in microglia [64]. In addition, in ASC-transplanted APP/PS1 mice, more microglial cells are present around these plaques and an increase of microglia-secreted Aβ degrading enzymes, such as IDE, NEP and MMP-9, is measured, compared with HBSS-treated control APP/PS1 mice. The switch in the microglial phenotype also causes a downregulation of the pro-inflammatory cytokines TNF-α and IL-1β, as well as an upregulation of arginase-1. This reduced neuroinflammation ultimately leads to improved cognitive abilities in ASC-transplanted mice, as measured by the Morris water maze task, a hippocampus-dependent test for spatial/memory brain functions [62].

ASC also significantly reduce neuroinflammation and ameliorate disease symptoms in PD [61,65]. Even six months after PD induction by LPS injection into SN and ASC transplantation, the number of pro-inflammatory microglia, marked by Ox-6, is significantly reduced in the SN, compared with LPS-injected animals without ASC transplantation, indicating the long-term beneficial effects of ASC. Moreover, dopaminergic neurons, detected with tyrosine hydroxylase (TH) staining and that die during the course of the disease, are recovered after ASC treatment [65].

Likewise, a beneficial impact of ASC is detected in other neurological pathologies, for example, in chronic mild stress (CMS), traumatic brain injury (TBI), ischemic stroke and spinal cord injury (SCI) (see Table 1). Similar to AD and PD, CMS is also characterized by microglial activation and the secretion of pro-inflammatory cytokines, such as TNF-α, IL-1β and IL-6 [7]. The intravenous injection of ASC into mice 3 weeks after CMS induction reduces the production of these cytokines. Furthermore, CMS-induced microglial pro-inflammatory activation, measured by the increase in Iba1-positive cells, is also downregulated. To demonstrate the neuroprotective effect of ASC, the authors measured the expression of BDNF and its receptor TrkB. While CMS reduces the expression of both proteins, treatment with ASC recovers their expression levels. Consequently, ASC administration reverses the depressive-like behavior induced by CMS [7], indicating that ASC exert their neuroprotective effects via microglia and that these molecular findings translate into the clinical improvement of this pathology.

For the treatment of TBI, three different reports used ASC-CM or ASC-CCM to ameliorate its clinical symptoms. While Xu and colleagues used a Morris water maze to demonstrate that ASC-CM-treated animals recover more quickly from TBI [57], Jha et al. measured visual acuity and contrast sensitivity, which were reduced as sequelae of mild TBI (mTBI) but posteriorly improved in mice treated with ASC-CCM [8,42]. In addition, these authors showed that the protein TSG-6, which is present in both ASC-CM and ASC-CCM [57], was at least partially responsible for this ameliorating effect [42]. Both groups also demonstrated that ASC-CM and ASC-CCM are able to downregulate neuroinflammation and microglial activation in vivo [8,42,57] as well as to recover neuronal survival [8,57].

Taken together, ASC and their CM are also able to induce a neuroprotective microglial phenotype in vivo. This provokes the downregulation of numerous neuroinflammatory indicators, improving clinical symptoms in animal models for a variety of neurological pathologies, as summarized in Table 1.

### 6.2. Application of ASC-Generated Extracellular Vesicles (EV) In Vivo

ASC transplantation has shown respectable results in certain animal models of neuropathologies, but the fact that just a small proportion of injected cells seems to differentiate into neurons suggests that the main protective effects are related to their paracrine activity [82]. In this sense, in addition to the factors present in the ASC secretome, the existence of EV should be taken into account. The release of these EV from different cell types has emerged as a new type of intercellular communication and it is proposed that they are responsible for the beneficial effects observed in stem cell therapy.

Mainly based on their size, cargo and route of release, EV are classified into different types, one of them being exosomes, approximately 30 to 120 nm-sized vesicles containing different proteins, lipids and nucleic acids [31]. The specific cargo can act locally or be transferred to recipient cells to mediate the functional effects on them [83]. In the brain, EV have been associated with neuron-glia crosstalk in order to preserve brain homeostasis, although they have also been related to the spreading of certain neurodegenerative diseases [84,85].

In the context of microglia, recent studies using exosomes released from neuroprotective microglia have revealed that they can modify the gene expression profile toward a neuroprotective phenotype in dysfunctional microglia and promote neural survival in different neuropathologies [86,87,88]. In addition, microglial EV also affect other cell types, e.g., EV from pro-regenerative microglia have been shown to promote oligodendrocyte differentiation [40,89], while EV released by microglia transduced with IL-4 ameliorate the disease outcome in experimental autoimmune encephalomyelitis (EAE), a mouse model for multiple sclerosis (MS) [90]. However, limitations to obtaining microglia-released exosomes diminish their direct application in the clinic.

On the contrary, ASC-derived exosomes can easily be isolated and stored in large amounts at a relatively low cost. The optimal administration for effective treatment is still controversial since only a small proportion of exosomes seems to pass the blood-brain barrier by classical intravenous injection [91], while they can reach the brain rapidly and efficiently via intranasal administration [66]. Proteomic and RNAseq analysis revealed that these exosomes are enriched in proteins and nucleic acids that promote neuroprotection and neurogenesis, as well as reducing inflammation [66,92]. Indeed, ASC-derived exosomes act in neurite remodeling and functional recovery after ischemic stroke [71] and TBI in rats [81]. They even reversed memory loss in AD mouse models [66]. These effects are similar to those induced by microglia-derived exosomes in recipient microglial cells [93]; accordingly, ASC- and BM-MSC-derived exosomes are mainly accumulated in microglia and, to a lesser extent, in astrocytes and neurons [81,94]. This points toward a microglia-mediated reduction of neuroinflammatory parameters, supported by studies that show that ASC-EV provoke a decrease in pro-inflammatory markers, such as CD68, inducible nitric oxide synthase (iNOS) and a variety of pro-inflammatory cytokines, and an increase in arginase-1 expression, switching microglia toward a neuroprotective phenotype with a more ramified cell shape [75,79,81].

In summary, ASC-derived exosomes alter the morphological characteristics of microglia, reduce significantly their pro-inflammatory activation during brain injury, and, consequently, decrease neuroinflammation to facilitate functional recovery. Therefore, ASC-derived exosomes may represent a cell-free therapy whose effect is mediated via communication with microglia, with a clear therapeutic potential for the treatment of a variety of neuropathologies in the future.

## 7. Conclusions and Outlook

In March 2018, the European Medicines Agency (EMA) authorized the first ASC-based therapy (called Alofisel, the only authorized medicament based on MSC so far) for the treatment of a complication related to an immune disorder, such as Crohn’s disease, due to the immunoregulatory and tissue-regenerative properties of these cells. This milestone verified several facts. First, it was the proof of concept of clinical translation of the safety and efficacy of ASC-based therapies found in preclinical models, and it opened the door to translating their use to other medical-clinical conditions with dysregulated immune responses and tissue damage, including various neuroinflammatory disorders. In this sense, it is evident that the use of a cell-based medicament that consists of a factory of several multi-modal immunomodulatory components, which might act synergistically, is an advantage versus therapies that are directed against a single mediator. Second, this authorization of the first ASC-based therapy confirmed the use of allogeneic ASC as a preferential source of MSC for future therapies. The “immune-privileged” status of ASC switched the previous syngeneic scenario of cell-based therapies, contingent on using autologous cells, to an allogeneic scenario that allows an off-the-shelf therapy, which is tremendously attractive for pharmaceutical companies and highly accessible for most health systems. This strategy solves a critical clinical problem of cell-based therapies, such as the necessity of isolating and expanding the MSC of the patient in good manufacturing practices (GMP) conditions to obtain a sufficient number of cells for immediate administration. Moreover, this aspect is especially important when treating patients with age-related disorders, such as neurodegenerative diseases, because the age of the donors is a major negative factor determining the lifespan and quality of MSC [95,96]. Especially in the case of older patients suffering neurodegeneration, allogeneic MSC from healthy young donors are clearly preferable to autologous MSC. Third, ASC show several advantages over other traditionally used types of MSC, like BM-MSC, as adipose tissue is more accessible, abundant and easier to obtain, e.g., by liposuction, than bone marrow. Further evidence indicates that, in comparison to BM-MSC, ASC show reduced susceptibility to NK cell-mediated lysis and perform improved immunomodulatory activities [97,98,99].

Future studies will also reveal if genetically modified MSC, secreting anti-inflammatory factors, will be even more effective than non-modified MSC [100]. For example, ASC transduced either with IL-4 [73] or the immunomodulatory neuropeptide vasoactive intestinal peptide (VIP) [74] show a higher efficacy in ameliorating the disease symptoms of the EAE mouse model for MS than non-modified ASC [73,74].

Finally, in our opinion, we will witness a significant growth of ASC-based therapies (either ASC themselves, their secretome or EV) in the next decade. Neurodegenerative disorders are very likely to benefit from this development, as ASC-based treatment of a variety of these pathologies is currently being examined in clinical trials due to their clear advantages, as stated above.

## Figures and Tables

**Table 1:** List of in vitro and in vivo studies using ASC to modulate microglia phenotypes and/or to ameliorate neurodegenerative diseases.

Disease	Experimental Model	ASC Administration	Publication Title	References
AD	APP/PS1 transgenic mouse	Intracerebral transplantation (hippocampus) of ASC	Intracerebral transplantation of adipose-derived mesenchymal stem cells alternatively activates microglia and ameliorates neuropathological deficits in Alzheimer’s disease mice	[62]
AD	APP/PS1 transgenic mouse	Intranasal administration of ASC-EV	ADSCs-derived extracellular vesicles alleviate neuronal damage, promote neurogenesis and rescue memory loss in mice with Alzheimer’s disease	[66]
AD	Intra-hippocampal injections of Aβ in rats	Intraperitoneal injections of ASC-CM	Hypoxic-conditioned medium from adipose tissue mesenchymal stem cells T improved neuroinflammation through alternation of toll-like receptor (TLR) 2 and TLR4 expression in a model of Alzheimer’s disease rats	[67]
CMS	CMS induction in mice	Intravenous injection of murine ASC 3 weeks after CMS induction	Adipose-derived mesenchymal stem cells protect against CMS-induced depression-like behaviors in mice via regulatingthe Nrf2/HO-1 and TLR4/NF-κB signaling pathways	[7]
Ischemic stroke	Acute ischemic stroke (AIS) model in rats	Intravenous injection of pig ASC and exosomes 3 h after AIS induction	Intravenous administration of xenogenic adipose-derived mesenchymal stem cells (ADMSC) and ADMSC-derived exosomes markedly reduced brain infarct volume and preserved neurological function in rats after acute ischemic stroke	[68]
Ischemic stroke	Transient global cerebral ischemia (GCI) model in rats	Intravenous injection of human ASC directly after induction of GCI	Adipose-derived mesenchymal stem cells reduce neuronal death after transient global cerebral ischemia through prevention of blood-brain barrier disruption and endothelial damage ^1^	[69]
Ischemic stroke	Transient GCI model in rats	Intravenous injection of human ASC	Effect of adipose-derived mesenchymal stem cell administration and mild hypothermia induction on delayed neuronal death after transient global cerebral ischemia	[70]
Ischemic stroke	Intracerebral injection of endothelin-1 to induce subcortical ischemic stroke	Intravenous injection of rat EV 24 h after stroke induction	White matter repair after extracellular vesicles administration in an experimental animal model of subcortical stroke ^1^	[71]
Ischemic stroke	Middle cerebral artery occlusion in rats	Intracerebral transplantation of rat ASC 8 days after ischemia induction	Immunological effects of the intraparenchymal administration of allogeneic and autologous adipose-derived mesenchymal stem cells after the acute phase of middle cerebral artery occlusion in rats ^1^	[72]
MS	EAE mouse model	Intraperitoneal injection of human allogenic ASC	Allogeneic adipose-derived mesenchymal stromal cells ameliorate experimental autoimmune encephalomyelitis by regulating self-reactive T cell responses and dendritic cell function	[6]
MS	EAE mouse model	Intraperitoneal administration of human allogenic ASC expressing IL-4 at disease onset	Early intervention with gene-modified mesenchymal stem cells overexpressing interleukin-4 enhances anti-inflammatory responses and functional recovery in experimental autoimmune demyelination	[73]
MS	EAE mouse model	Intraperitoneal administration of mouse allogenic ASC expressing VIP at the peak of disease	Mesenchymal stem cells expressing vasoactive intestinal peptides ameliorate symptoms in a model of chronic multiple sclerosis.	[74]
MS	EAE mouse model	Intravenous administration of ASC before and after disease onset	Adipose-derived mesenchymal stem cells ameliorate chronic experimental autoimmune encephalomyelitis ^1^	[56]
MS	MS model of Theiler’s murine encephalomyelitis virus (TMEV)	Intravenous injection of ASC-EV on day 60 postinfection	Therapeutic potential of extracellular vesicles derived from human mesenchymal stem cells in a model of progressive multiple sclerosis	[75]
Neuro-inflammation	BV2 cells	ASC-BV2 cells co-culture	Adipose-derived mesenchymal stem cells protect against CMS-induced depression-like behaviors in mice via regulating the Nrf2/HO-1 and TLR4/NF-κB signaling pathways	[7]
Neuro-inflammation	BV2 cells	Microglia incubated with ASC-CCM	Concentrated conditioned media from adipose tissue-derived mesenchymal stem cells mitigates visual deficits and retinal inflammation following mild traumatic brain injury	[8]
Neuro-inflammation	BV2 cells	Microglia incubated with ASC-CCM	TSG-6 in conditioned media from adipose mesenchymal stem cells protects against visual deficits in mild traumatic brain injury model through neurovascular modulation	[42]
Neuro-inflammation	Primary mouse microglia	Microglia with ASC plated in transwells, microglia incubated with ASC-CM	Mesenchymal stem cells induce the ramification of microglia via the small RhoGTPases Cdc42 and Rac1	[22]
Neuro-inflammation	Primary mouse microglia	Microglia incubated with ASC-CM	The atypical RhoGTPase RhoE/Rnd3 is a key molecule to acquire a neuroprotective phenotype in microglia	[54]
Niemann-Pick disease type C	Niemann–Pick disease type C model mice	Transplantation of ASC in mouse cerebellum	Adipose tissue-derived stem cells rescue Purkinje neurons and alleviate inflammatory responses in Niemann-Pick disease type C mice ^1^	[76]
PD	Intrastriatal 6-hydroxydopamine injections of rats	Intracerebral transplantation (SN) of human ASC	Human adipose-derived mesenchymal stem cells improve motor functions and are neuroprotective in the 6-hydroxydopamine-rat model for Parkinson’s disease when cultured in monolayer cultures but suppress hippocampal neurogenesis and hippocampal memory function when cultured in spheroids ^1^	[77]
PD	Intrastriatal 6-hydroxydopamine injections of mice	Intracerebral transplantation (SN) of ASC one week after the 6-hydroxydopamine injections	Autologous transplants of adipose-derived adult stromal (ADAS) afford dopaminergic neuroprotection in a model of Parkinson’s disease ^1^	[61]
PD	LPS-injection into SN	Intracerebral transplantation (SN) of ASC at the same time as LPS injection	Adipose-derived stem cells decreased microglial activation and protected dopaminergic loss in a rat lipopolysaccharide model	[65]
PD	Intrastriatal 6-hydroxydopamine injections of rats	Intracerebral transplantation (SN) of human ASC	Human adipose-derived mesenchymal stromal cells increase endogenous neurogenesis in the rat subventricular zone acutely after 6-hydroxydopamine lesioning ^1^	[78]
Retinal inflammation following mTBI	mTBI mouse model	Intravitreal injections of ASC-CCM	Concentrated conditioned media from adipose tissue-derived mesenchymal stem cells mitigates visual deficits and retinal inflammation following mild traumatic brain injury	[8]
Retinal inflammation following mTBI	mTBI mouse model	Intravitreal injections of ASC-CCM	TSG-6 in conditioned media from adipose mesenchymal stem cells protects against visual deficits in mild traumatic brain injury model through neurovascular modulation	[42]
SCI	SCI model in mice	Intravenous injection of ASC-EV immediately after SCI induction	Exosomes from long noncoding RNA-Gm37494-ADSCs repair spinal cord injury via shifting microglial M1/M2 polarization	[79]
SCI	Moderate contusion injury of the spinal cord in mice	Injection of ASC into SCI epicenter directly after SCI induction	Adipose mesenchymal stem cell transplantation alleviates spinal cord injury-induced neuroinflammation partly by suppressing the Jagged1/Notch pathway ^1^	[80]
TBI	TBI rat model	Intra-cerebroventricular injection of human ASC-EV	MSC-derived exosomes promote recovery from traumatic brain injury via microglia/macrophages in rat	[81]
TBI	TBI rat model	Intravenous injection of CM from human ASC after TBI	Intravenously infusing the secretome of adipose-derived mesenchymal stem cells ameliorates neuroinflammation and neurological functioning after traumatic brain injury	[57]

^1^ No experimental data with microglia included in this article. Abbreviations used in the table: AD, Alzheimer´s disease; ASC, adipose tissue-derived mesenchymal stem cells; AIS, acute ischemic stroke; ASC-CM, Conditioned medium from ASC; ASC-CCM, concentrated ASC-CM; CMS, chronic mild stress; EAE, experimental autoimmune encephalomyelitis; ASC-EV, extracellular vesicles from ASC; GCI, global cerebral ischemia; MS, multiple sclerosis; mTBI, mild traumatic brain injury; PD, Parkinson´s disease; SCI, spinal cord injury; SN, Substantia Nigra; TBI, traumatic brain injury; TMEV, MS model of Theiler’s murine encephalomyelitis virus.

## Data Availability

All data presented in this study is contained within this article and its supplementary material. Raw data are available upon reasonable request to the corresponding author.

## Supplementary Materials

The following are available online at https://www.mdpi.com/article/10.3390/biom12020219/s1, Video S1: Ramification of microglia upon administration of conditioned medium from adipose tissue-derived mesenchymal stem cells (ASC-CM); Video S2: Lamellipodia formation upon administration of conditioned medium from adipose tissue-derived mesenchymal stem cells (ASC-CM). Supplementary File S3: Supplementary legends to Video S1 and Video S2.

## Abbreviations

ADNP	activity-dependent neurotrophic protein
Aβ	β-amyloid peptide
AD	Alzheimer´s disease
AIS	acute ischemic stroke
APP	Alzheimer Aβ precursor protein
ASC	adipose tissue-derived mesenchymal stem cells
ASC-CCM	concentrated conditioned medium from ASC
ASC-CM	conditioned medium from ASC
ASC-EV	extracellular vesicles from ASC
BDNF	brain-derived neurotrophic factor
BM-MSC	bone marrow-derived MSC
CM	conditioned medium
CMS	chronic mild stress
CNS	central nervous system
EAE	experimental autoimmune encephalomyelitis
EMA	European Medicines Agency
EV	extracellular vesicles
FGF-2	fibroblast-growth factor-2
GCI	global cerebral ischemia
GMP	good manufacturing practices
HLA	human leucocyte antigen
HO-1	heme oxygenase-1
IFN-γ	interferon-γ
IL	Interleukin
iNOS	inducible nitric oxide synthase
LPS	lipopolysaccharide
MS	multiple sclerosis
MSC	mesenchymal stem cells
mTBI	mild traumatic brain injury
NK	natural killer cells
NO	nitric oxide
PD	Parkinson´s disease
PS1	Presilin1
PI3K	phosphoinositol-3-kinase
PKB	protein kinase B/Akt
PIP3	phophoinositol-3-phosphate
SCI	spinal cord injury
SN	substantia nigra
TBI	traumatic brain injury
TH	tyrosine hydroxylase
TMEV	MS model of Theiler’s murine encephalomyelitis virus
TNF-α	tumor necrosis factor-α
RhoGEF	Rho guanine nucleotide exchange factor
VIP	vasoactive intestinal peptide
