# Supplementary material for: Switching Roles: Beneficial Effects of Adipose Tissue-Derived Mesenchymal Stem Cells on Microglia and Their Implication in Neurodegenerative Diseases
Source: Biomolecules. 2022 Jan 27;12(2):219. doi: 10.3390/biom12020219 (PMC8961583; doi:10.3390/biom12020219)
Supplement: Supplementary file 1 [file biomolecules-12-00219-s001.zip › biomolecules-1558297 -Supplementary File S3.pdf]

Supplementary File S3: Supplementary legends to Video S1 and Video S2

Supplementary Video S1: Ramification of microglia upon administration of conditioned medium from adipose tissue-derived mesenchymal stem cells (ASC-CM). Primary murine microglia were plated in a 12-well plate in microglia growth medium [22] and subjected to live cell imaging, as described in the legend of Figure 2C. The time lapse was terminated after 8 h and the video was created using the Image analysis software ImageJ/FiJi (NIH, USA) at 5 frames per second.

Supplementary Video S2: Lamellipodia formation upon administration of conditioned medium from adipose tissue-derived mesenchymal stem cells (ASC-CM). Primary murine microglia were plated in a 12-well plate in microglia growth medium [22] and subjected to live cell imaging, as described in the legend of Figure 2C. Immediately after the 0 min time point, ASC-CM was added to the cells and pictures were taken every min from the same field of view. The time lapse was terminated after 60 min and the video was created using the Image analysis software ImageJ/FiJi (NIH, USA) at 5 frames per second. Note: The upper-left cell phagocytosed cell debris from the tissue culture medium and the phagosome is visible as white spot inside the cell.
